# Bisphosphates for Osteoporosis: A Bibliometric Analysis of the Most Cited Articles

**DOI:** 10.1155/2022/4565069

**Published:** 2022-05-18

**Authors:** Yu Zhang, Man Hu, Lei Zhu, Junwu Wang, Pingchuan Wang, Pengzhi Shi, Wenjie Zhao, Xin Liu, Qing Peng, Bo Meng, Chen Chen, Xinmin Feng, Yongxiang Wang, Liang Zhang

**Affiliations:** ^1^Department of Orthopedics, Clinical Medical College of Yangzhou University, Yangzhou 225001, China; ^2^Department of Orthopedics, Dalian Medical University, Dalian 116000, China; ^3^Graduate School of Xuzhou Medical University, Xuzhou 221004, China

## Abstract

Osteoporosis has become a major public health problem and bisphosphates treatment for osteoporosis is a rapidly developing research field. Every year, plenty of studies devoted to the treatment of osteoporosis are published, giving clinicians a new perspective on bisphosphates treatment for osteoporosis. However, the quality of the scientific papers in this area is unclear. The aim of the present study was to characterize the 100 top-cited articles regarding bisphosphates treatment for osteoporosis. This analysis provides an accessible list for practitioners of endocrinology, pharmacy, epidemiology, imaging, surgery, and scientific research to identify the most frequently cited literature and better understand the future direction.

## 1. Introduction 

Osteoporosis is a common metabolic bone disease, which is related to the decrease of bone mineral density (BMD) and bone mass and increase of bone fragility [1–3]. The distribution of osteoporosis is different in different populations. In the United States, 10 million people over the age of 50 suffer from osteoporosis, and another 34 million people are at risk [4]. It is reported that the incidence of osteoporosis in Europe in 2010 was 27.6 million [5]. According to the national statistical yearbook, the number of people aged over 60 in China has exceeded 210 million. By the end of 2015, the number of elderly patients with osteoporosis has exceeded 75 million [6]. The most serious consequence of osteoporosis is fracture, which causes a significant economic burden on health care systems around the world [7, 8].

At present, the drug treatment for osteoporosis can be divided into two categories: anti-resorption drugs which slow down bone resorption, and synthetic metabolic drugs which stimulate bone formation [9]. Bisphosphonates are the backbone of anti-resorption drugs, which show high affinity to bone and long-term safety and can be taken orally or intravenously [10–12]. Bisphosphates binds to the free hydroxyapatite that can be obtained on the bone surface, has a strong affinity with bone tissue, and resists enzyme digestion. In the process of bone resorption, bisphosphate will be desorbed from hydroxyapatite and absorbed by osteoclasts, reducing the metabolic activity of osteoclasts, weakening the bone resorption capacity of osteoclasts, preventing osteoblasts and osteocytes from apoptosis, and increasing the number and function of osteoblasts [13, 14]. The anti-apoptotic effect of bisphosphates is separated from the effect of drugs on osteoclasts, protecting the bone formation function of mature osteoblasts and maintaining the osteocyte network, coupled with the lack of anti-catabolism. Thus, bisphosphonates are the most widely used anti-resorption drugs for osteoporosis mainly in post-menopausal women. Every year, plenty of studies devoted to the treatment of osteoporosis are published, giving clinicians a new perspective on bisphosphates treatment for osteoporosis. The quality of scientific studies in this field, however, is uncertain. Identifying the most influential pieces remains a difficulty.

The citation can be used as an indicator of the scientific influence of an article in its field [15, 16]. Bibliometric analysis is an important tool to help quantify the number of articles in disciplines and provide a comprehensive overview of the literature [17, 18]. Bibliometric analysis is now widely used in many disciplines, including anesthesiology [19], orthopedics [20], endocrinology [21], oncology [22], obstetrics, and gynecology [23].

The purpose of this study was to identify and analyze the 100 most-cited literature related to bisphosphate treatment for osteoporosis through extensive literature search methods. Appreciating and learning from these influential publications can help clinicians better make treatment decisions and understand the future direction.

## 2. Materials and Methods

Our study was a retrospective assessment of the public data, so the approval of the institutional review committee is not required. A bibliometric analysis of bisphosphates treatment for osteoporosis was performed on August 5th, 2021, using the Web of Science (WOS). No restrictions were placed on country of origin or medical specialty. As in other documents, England, Wales, Northern Ireland, and Scotland were classified as the United Kingdom. First, osteoporosis and bisphosphate were used as search terms. To ensure the breadth and relevance of the search scope, the keywords were constantly filtered. And then, the final set of search terms were ‘osteoporosis' AND (‘bisphosphonate' or ‘BPs' OR ‘alendronate' or ‘risedronate' or ‘etidronate' or ‘ibandronate' or ‘clodronate' or ‘pamidronate' or ‘zoledronate' or ‘zoledronic acid') included in the “Title” search. The search results were sorted by the times cited in the WOS, and the 100 most-cited articles on bisphosphates for osteoporosis were derived. Again, the search time was set from 2019 to 2021, and the search results were sorted by the times cited in the WOS; the 50 most cited articles on bisphosphates for osteoporosis were derived. The citations per year index (CPYI) was introduced to eliminate the tendency of older articles to accumulate more citations. Two independent authors (Zhang and Hu) performed the search, screened the articles, and extracted the information. The third author (Zhang) was consulted if differences arise. The number of citations, authorship, journal of publication, year of publication, and country were extracted. VOS viewer software 1.6.16 (Van Eck and Waltman, Leiden University, Leiden, The Netherlands) was used for network visualization analysis of keywords [24]. The terms are used more frequently as when the circle grows larger.

## 3. Results

### 3.1. Characteristics of the Top 100 Most-Cited Articles

The searching results identified 2761 articles on bisphosphates for the treatment of osteoporosis in the WOS database. The 100 most-cited articles were published from 1976 to 2018, and the number of citations ranged from 102 to 1884. These articles were collectively cited 31325 times at the time of the search. The annual citations index ranged from 2.26 to 123.67. The most highly cited article was “Effect of Oral Alendronate on Bone Mineral Density and the Incidence of Fractures in Post-Menopausal Osteoporosis” published in New England Journal of Medicine by Liberman, Uri A et al. This article ranked fourth in annual citations (69.78 per year). Of the top 10 articles in terms of total citations, 5 articles were published in the New England Journal of Medicine. Four articles were cited more than 1000 times and 15 articles were cited more than 500 times (Table 1).

### 3.2. Analysis of Country and Publishing Trend

**Figure 1:**
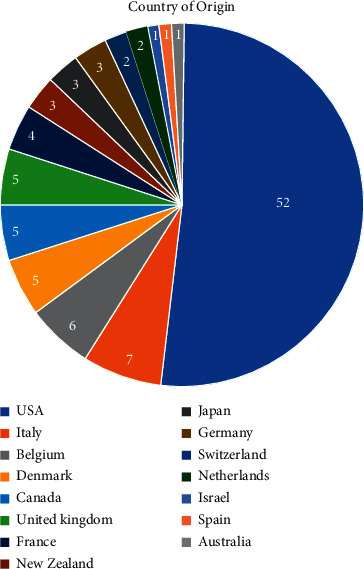
The top 100-cited articles on bisphosphates for osteoporosis based on country.

According to the affiliation of the first author, the 100 most-cited articles in bisphosphates for osteoporosis were from 16 countries, with most publications from the United States (52 articles), followed by Italy (7 articles), Belgium (6 articles), Denmark, United Kingdom and Canada (5 articles each), France (4 articles), New Zealand, Japan, and Germany (3 articles each), and Switzerland and Netherlands (2 articles each). The remaining countries (Israel, Spain, and Australia) published one article each in the top 100 cited list (Figure 1).

All the 100 most-cited articles were published between 1976 and 2018. The most productive periods were 2000 to 2009, with a total of 53 articles, followed by 1990–1999, 2010–2019, 1980–1989, and 1970–1979 with 27, 17, 2, and 1, respectively. Heaney et al. published the first article in 1976, reporting Etidronate as a strong bone remodeling inhibitor. The most recent article was published by Kendler et al. in 2018, describing a double-blind randomized controlled trial of teriparatide and risedronate in the treatment of new fractures in severe post-menopausal osteoporosis.

### 3.3. Journal Analysis

Twenty-nine journals contributed to the top 100 articles with 12 contributing two or more articles (Table 2). The most published journals were the New England Journal of Medicine and Journal of Bone and Mineral Research, with 16 articles, respectively. Moreover, the total number of citations and impact factors in the New England Journal of Medicine was also the highest. The Journal of Clinical Endocrinology and Metabolism ranked second with 14 articles.

### 3.4. Highly Contributive Authors

According to the ranked of the total number of co-authors published, 20 authors contributed 5 or more of the top 100 papers. Miller PD published the most articles, with 15 items. The main contributors to an article are usually the first and the last authors. Among the first authors, Black, DM published the most articles (*n* = 7), and the last author Delmas, PD published the most articles (*n* = 4) (Table 3).

### 3.5. Analysis of Study Types, Patients, Drugs, and Complications

The 100 most-cited articles were categorized as original studies (*n* = 89), systematic review/meta-analysis (*n* = 5), reviews (*n* = 5), and editorial material (*n* = 1). The treatment patients mainly included post-menopausal women and glucocorticoid-induced patients. Alendronate and risedronate were the two most used drugs. Four articles were on complications, including osteonecrosis of the jaw (*n* = 3) and upper gastrointestinal disorders (*n* = 1) (Table 4).

### 3.6. Keyword Analysis

**Figure 2:**
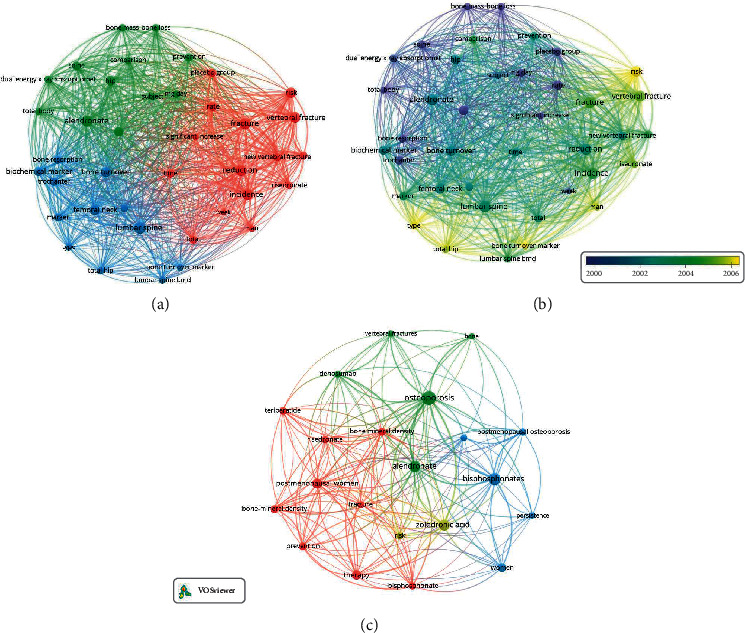
Keyword analysis. (a, c) Network visualization map showing cluster analysis of keywords associated with bisphosphates for osteoporosis. The larger the circle is, the more frequently the words are used. The curves between the nodes represent the co-occurrence of the two keywords. (b) Network visualization map showing the evolution of keyword frequency over time. Colors are assigned according to the average year in which keywords appeared in articles. Keywords in blue appeared earlier than those in yellow.

The keyword analysis is one of the most important indicators of bibliometrics. Co-occurrence analysis found that the relationship of items is based on the number of publications in which they occur together [25]. The co-occurrence network analysis tool was used to set the minimum number of occurrences to 12. A total of 39 keywords were included, which can be divided into 3 categories: “clinical study” with red, “drug and imagological study” with green, and “basic research” with blue. In the clinical study, the most popular keywords were “fracture (*n* = 41)”, “incidence (*n* = 37)”, “reduction (*n* = 35)”, “risk (*n* = 34)”, “vertebral fracture (*n* = 33)”, “new vertebral fracture (*n* = 21)”, and “significant increase (*n* = 14)”. The frequency of the keyword “fracture” was high across the years of the search period. In the drug and imagological study, the most popular keywords were “alendronate (*n* = 37)”, “decrease (*n* = 27)”, “hip (*n* = 25)”, “comparison (*n* = 17)”, “bone mass (*n* = 16)”, “bone loss (*n* = 15)”, and “Dual X-ray absorptiometry (DXA) (*n* = 15)”. In the basic research, the most popular keywords were “lumbar spine (*n* = 40)”, “bone turnover (*n* = 29)”, “biomedical markers (*n* = 24)”, “marker (*n* = 20)”, and “bone turnover marker (*n* = 14)” (Figure 2(a)).

To further determine the change of research topic over time, the evolution of the highest frequency keywords was evaluated using VOS viewer (Figure 2(b)). The blue color means the keyword appears early and yellow-colored keywords appear later. In the early stage of bisphosphate therapy for osteoporosis, most studies focused on “drug and imaging examination of bone mineral density”. The latest trends indicated that “basic research” on bone turnover markers and “clinical fracture and risk” clusters may receive widespread attention in the future.

### 3.7. The Most Cited Papers and Keyword Analysis in the Recent 2 Years

The most productive period was 2019 (*n* = 30) and the most published author was Sugimoto T (*n* = 4). The United States published (*n* = 10) the most articles, followed by Japan (*n* = 9) and China (*n* = 7). According to the network co-occurrence analysis of keywords, it was found that osteoporosis (*n* = 24) was the most common keyword, followed by bisphosphate (*n* = 18), zoledronic acid (*n* = 16), and alendronate (*n* = 15) (Figure 2(c)).

## 4. Discussion

Osteoporosis has become a major public health problem and bisphosphates treatment for osteoporosis is a rapidly developing research field. Bibliometrics analysis is a form of statistical analysis of published papers. Although it is not the only symbol of the scientific quality of an article, it can be used to quantify the citation frequency of a paper and be used as an alternative sign of influence in its field [17, 18, 26]. The present study was the first to conduct a bibliometric and keyword co-occurrence analysis of the 100 most influential articles on bisphosphates treatment for osteoporosis. At the same time, we also studied the bibliometric analysis of the most frequently cited latest literature in the recent 2 years to understand the current trends and hotspots. The results of this study may help to collate data and easily obtain the highest yield data of bisphosphates treatment for osteoporosis, thus helping clinicians better make a treatment decision and understand the future direction of this discipline.

The 100 most-cited articles published from 1976 to 2018 were cited 102 to 1884 times. The list of the articles identified topics that reflect changing trends in bisphosphates treatment for osteoporosis over the past 42 years. Although it was impossible to analyze all 100 highly cited articles in detail, we can find some important results. We found that the most cited publications in the field were mainly from the United States. The United States published 52 articles, followed by Italy and Belgium. The top countries are mainly distributed in North America and Europe, which are all developed countries. Thus, there is still a significant gap in the output of articles between developed countries and developing countries.

We found that the top 100 most-cited articles were mainly published before 2018. Through the further time limit of 2019–2021, there was no doubt that the country with the most published papers was still the United States, but the difference was that the second and third most cited publications in this field were Japan and China. This was roughly the same as the citation analysis of articles related to post-menopausal women with osteoporosis in the top countries [27, 28]. Similarly, in the dental and osteoporosis analysis studied by Qiu et al. Japan was found to be the most frequently cited publication country in this field [29]. The principles and concepts of anti-osteoporosis treatment in developed countries have been fully affirmed and established based on scientific research. In many aspects, it showed that China and Japan were two Asian countries that attach importance to the study of osteoporosis and become important participants in the treatment of osteoporosis. Bisphosphate therapy for osteoporosis will continue to attract medical researchers around the world to promote further research on the treatment of osteoporosis and bring new perspectives in this regard.

In terms of the most published journals, we found that the New England Journal of Medicine and Journal of Bone and Mineral Research are the most productive journals with the highest average number of citations. These results are in line with Bradford's law as if the researchers deviate from these core journals, their citation frequency will decrease [30]. Thus, researchers tend to cite papers from several core journals in their professional fields.

As is known to all, the date of publication can affect the number of citations. Older studies may have a higher number of citations and the recently released articles still need time to be cited widely. Previous citation analysis found that most of the first 100 cited papers were published from 1991 to 2000 [31, 32]. But in this analysis, the period from 2000 to 2009 was the most frequently cited period, with more than half of the articles. One possible explanation was that the intensification of global aging may lead to an increased incidence of osteoporosis [33, 34]. The other important reason might be the popularity of bisphosphate therapy and the promotion of imaging methods and basic research [8].

Among the multi-author papers, the first and the last authors usually contributed the most. To better clarify the contribution of researchers in this field, we collected the number of papers that the author participated in and counted the number of papers published by the first and the last authors. Miller PD was the co-author of all 15 articles, making him the most published researcher in the top 100. Dr. Miller and his team put more emphasis on individualized treatment which is found as effective as daily medication based on patients' compliance. Black, DM was another pioneer of bisphosphates for osteoporosis research who had the most first-author publications (*n* = 7). By analyzing the main authors in this field, we can identify the main contributors and look for opportunities for further cooperation.

The keyword co-occurrence analysis found that the most popular keywords were fracture, lumbar spine, alendronate, and incidence. Individuals with osteoporosis are at an increased risk of fragility fracture [35]. The fracture site often occurs in the lumbar vertebrae [36, 37]. Thus, routine DXA examination of lumbar BMD is necessary for osteoporosis patients in the clinical diagnosis. For the prevention, post-menopausal osteoporosis women and glucocorticoid-dependent osteoporosis patients routinely take bisphosphate drugs after being diagnosed by BMD examination. Bisphosphonates can significantly increase BMD of the whole body such as spine and hip and thus significantly reduce the risk ratio of a new spine fracture.

The visual map drawn by the VOS viewer was used to find the changing trend of keywords with time. A total of 39 included keywords was divided into clinical study, drug and imaging study, and basic research. In the initial study, we found that the study focuses gradually shifted from x-ray diagnosis and treatment to basic research, which was consistent with the trend reported by Qiu et al. [27]. By grasping the changing trend in this field, we can reduce resource waste and better understand the future direction.

Early keyword co-occurrence analysis found that alendronate was the most frequently used in clinical use. According to the most citations of 50 articles in recent 2 years, zoledronic acid was used most frequently, which was consistent with the results of Gao et al. [38]. After long-term bisphosphonate medication, patients with osteoporosis will re-evaluate the appropriate response. The curative impact will be influenced by patient compliance and varying drug absorption. The research trend shows that more and more studies turn to basic research, and more drugs may be used to treat osteoporosis in the future.

Timely bibliometrics and global analysis of all bisphosphates treatment for osteoporosis not only reveal the main countries, authors, and research impacts of the study but also provide information on the main research directions and trends, which will enable researchers to better understand the future direction. Predicting the fracture risk of osteoporosis patients in the future is still critical. More research into the usage of bisphosphonates in combination with other anti-osteoporosis treatments is needed in the future. Future study will focus on determining how to make the best use of existing treatments and develop better drugs.

### 4.1. Limitation

Although bibliometrics is an effective method to evaluate article influence, there are still several limitations in our current research. First, only WOS was used to search the literature, not the existing Google academic, Medline, or other databases [39]. The number of citations of the report may be slightly different. Second, the main language of WOS is English, which may lead to the omission of relevant articles in other countries [40]. Third, the number of citations may be higher for the older research, but the older articles may not keep up with current research hotspots [41, 42]. Newly published articles need more time to accumulate citations, and their influence may be underestimated [43]. With the continuous updating of the database, the bibliometrics analysis data may be different from the actual research situation, but the overall trend does not change much. Finally, one of the reasons for a high number of citations may be self-citation, including authors citing their own articles and authors citing more articles from the journals they want to publish [44]. Further research is needed to analyze the frequency of self-citation and its influence on the article. Despite these limitations, bibliometric analysis remains a valuable method for quantifying the number of articles published in various fields and providing a comprehensive overview of the literature. Our study is the first bibliometric analysis of bisphosphate for the treatment of osteoporosis. Moreover, our analysis can help clinicians better make treatment decisions and understand the future direction.

## 5. Conclusion

In summary, our study provides a detailed list and characteristics of the first 100 articles on bisphosphate for the treatment of osteoporosis. This analysis provides an accessible list for practitioners of endocrinology, pharmacy, epidemiology, imaging, surgery, and scientific research to identify the most frequently cited literature and better understand the future direction.

## Figures and Tables

**Table 1:** The 100 most-cited articles on bisphosphates for osteoporosis.

Rank	Title	First author	Publishing year	Journal	Cited times	Citation/year
1	Effect of Oral Alendronate on Bone Mineral Density and the Incidence of Fractures in Post-Menopausal Osteoporosis	Liberman, Uri A	1995	New England Journal of medicine	1884	69.78 (4)
2	Once-Yearly Zoledronic Acid for Treatment of Post-Menopausal Osteoporosis	Black, DM	2007	New England Journal of medicine	1855	123.67 (1)
3	Effects of Risedronate Treatment on Vertebral and Non-Vertebral Fractures in Women with Post-Menopausal Osteoporosis - A Randomized Controlled Trial	Harris, ST	1999	Jama-Journal of the American Medical Association	1829	79.52 (2)
4	Randomized Trial of the Effects of Risedronate on Vertebral Fractures in Women with Established Post-Menopausal Osteoporosis	Reginster, JY	2000	Osteoporosis International	1179	53.59 (6)
5	Ten Years' Experience with Alendronate for Osteoporosis in Post-Menopausal Women	Bone, HG	2004	New England Journal of Medicine	995	55.28 (5)
6	Alendronate for the Prevention and Treatment of Glucocorticoid-Induced Osteoporosis	Saag, Kenneth G	1998	New England Journal of Medicine	913	38.04 (11)
7	Effects of Oral Ibandronate Administered Daily or Intermittently on Fracture Risk in Post-Menopausal Osteoporosis	Chesnut, CH	2004	Journal of Bone and Mineral Research	891	49.5 (7)
8	Intermittent Cyclical Etidronate Treatment of Post-Menopausal Osteoporosis	Watts, N B	1990	The New England Journal of Medicine	879	27.47 (18)
9	The Effects of Parathyroid Hormone and Alendronate Alone or in Combination in Post-Menopausal Osteoporosis	Black, DM	2003	New England Journal of Medicine	866	45.58 (9)
10	Effect of Intermittent Cyclical Etidronate Therapy on Bone Mass and Fracture Rate in Women with Post-Menopausal Osteoporosis	Storm, T	1990	The New England Journal of Medicine	846	26.44 (19)
11	Fracture Risk Reduction with Alendronate in Women with Osteoporosis: The Fracture Intervention Trial	Black, DM	2000	Journal of Clinical Endocrinology and Metabolism	744	33.82 (14)
12	Alendronate for the Treatment of Osteoporosis in Men	Orwoll, E	2000	New England Journal of Medicine	650	29.55 (17)
13	Intermittent Etidronate Therapy to Prevent Corticosteroid-Induced Osteoporosis	Adachi, Jonathan D	1997	New England Journal of Medicine	594	23.76 (22)
14	The Effects of Parathyroid Hormone, Alendronate, or Both in Men with Osteoporosis	Finkelstein, JS	2003	New England Journal of Medicine	580	30.53 (15)
15	Teriparatide or Alendronate in Glucocorticoid-Induced Osteoporosis	Saag, Kenneth G	2007	New England Journal of Medicine	543	36.2 (12)
16	Histomorphometric Assessment of the Long-Term Effects of Alendronate on Bone Quality and Remodeling in Patients with Osteoporosis	Chavassieux, Pascale M	1997	Journal of Clinical Investigation	436	17.44 (32)
17	One Year of Alendronate after One Year of Parathyroid Hormone (1–84) for Osteoporosis	Black, DM	2005	New England Journal of Medicine	431	25.35 (20)
18	Efficacy and Safety of Daily Risedronate in the Treatment of Corticosteroid-Induced Osteoporosis in Men and Women: A Randomized Trial	Reid, DM	2000	Journal of Bone and Mineral Research	408	18.55 (28)
19	Romosozumab or Alendronate for Fracture Prevention in Women with Osteoporosis	Saag, Kenneth G	2017	New England Journal of Medicine	391	78.2 (3)
20	Therapeutic Equivalence of Alendronate 70 mg Once-Weekly and Alendronate 10 mg Daily in the Treatment of Osteoporosis	Schnitzer, T	2000	Aging Clinical and Experimental Research	370	16.82 (33)
21	The Effect of 3 versus 6 Years of Zoledronic Acid Treatment of Osteoporosis: A Randomized Extension to the HORIZON-Pivotal Fracture Trial (PFT)	Black, DM	2012	Journal of Bone and Mineral Research	346	34.6 (13)
22	Meta-Analysis of Alendronate for the Treatment of Post-Menopausal Women	Cranney, A	2002	Endocrine Reviews	332	16.6 (34)
23	Prevention of Steroid-Induced Osteoporosis with (3-Amino-1-hydroxypropylidene)-1,1-bisphosphonate (APD	Reid, IR	1988	Lancet	331	9.74 (56)
24	Four-Year Study of Intermittent Cyclic Etidronate Treatment of post-Menopausal Osteoporosis: Three Years of Blinded Therapy Followed by One Year of Open Therapy	Harris, ST	1993	The American Journal of Medicine	324	11.17 (50)
25	Compliance and Persistence with Bisphosphonate Dosing Regimens among Women with Post-Menopausal Osteoporosis	Cramer, JA	2005	Current Medical Research and Opinion	321	18.88 (27)
26	Zoledronic Acid and Risedronate in the Prevention and Treatment of Glucocorticoid-Induced Osteoporosis (HORIZON): A Multicentre, Double-Blind, Double-Dummy, Randomized Controlled Trial	Reid, David M	2009	Lancet	311	23.92 (21)
27	Managing Osteoporosis in Patients on Long-Term Bisphosphonate Treatment: Report of a Task Force of the American Society for Bone and Mineral Research	Adler, Robert A	2016	Journal of Bone and Mineral Research	295	49.17 (8)
28	Effects of Teriparatide versus Alendronate for Treating Glucocorticoid-Induced Osteoporosis Thirty-Six-Month Results of a Randomized, Double-Blind, Controlled Trial	Saag, Kenneth G	2009	Arthritis and Rheumatism	282	21.69 (24)
29	Bisphosphonate Therapy for Osteoporosis: Benefits, Risks, and Drug Holiday	McClung, Michael	2013	American Journal of Medicine	274	30.44 (16)
30	Skeletal Benefits of Alendronate: 7-Year Treatment of Post-Menopausal Osteoporotic Women	Tonino, RP	2000	Journal of Clinical Endocrinology and Metabolism	270	12.27 (46)
31	Treatment with Once-Weekly Alendronate 70 mg Compared with Once-Weekly Risedronate 35 mg in Women with Post-Menopausal Osteoporosis: A Randomized Double-Blind Study	Rosen, CJ	2005	Journal of Bone and Mineral Research	268	15.76 (37)
32	Monthly Oral Ibandronate Therapy in Post-Menopausal Osteoporosis: 1-Year Results from the MOBILE Study	Miller, PD	2005	Journal of Bone and Mineral Research	264	15.53 (38)
33	A Randomized Double-Blind Trial to Compare the Efficacy of Teriparatide [Recombinant Human Parathyroid Hormone (1–34)] with Alendronate in Post-Menopausal Women with Osteoporosis	Body, JJ	2002	Journal of Clinical Endocrinology and Metabolism	257	12.85 (44)
34	Seven Years of Treatment with Risedronate in Women with Post-Menopausal Osteoporosis	Mellstrom, DD	2004	Calcified Tissue International	245	13.61 (41)
35	Efficacy and Tolerability of Once-Monthly Oral Ibandronate in Post-Menopausal Osteoporosis: 2-Year Results from the MOBILE Study	Reginster, JY	2006	Annals of the Rheumatic Diseases	244	15.25 (39)
36	The Efficacy and Tolerability of Risedronate Once a Week for the Treatment of Post-Menopausal Osteoporosis	Brown, JP	2002	Calcified Tissue International	233	11.65 (48)
37	Larger Increases in Bone Mineral Density during Alendronate Therapy Are Associated with a Lower Risk of New Vertebral Fractures in Women with Post-Menopausal Osteoporosis	Hochberg, MC	1999	Arthritis and Rheumatism	232	10.09 (54)
38	Prevention of Nonvertebral Fractures by Alendronate. A Meta-Analysis. Alendronate Osteoporosis Treatment Study Groups	Karpf, DB	1997	JAMA	232	9.28 (60)
39	Alendronate Prevents Post-Menopausal Bone Loss in Women without Osteoporosis: A Double-Blind, Randomized, Controlled Trial	Mcclung, Michael	1998	Annals of Internal Medicine	221	9.21 (62)
40	Effects of Intravenous Zoledronic Acid plus Subcutaneous Teriparatide [rhPTH(1–34)] in Postmenopausal Osteoporosis	Cosman, felicia	2011	Journal of Bone and Mineral Research	217	19.73 (25)
41	Meta-Analysis of Risedronate for the Treatment of Post-Menopausal Osteoporosis	Cranney, A	2002	Endocrine Reviews	217	10.85 (51)
42	Intravenous Ibandronate Injections in Post-Menopausal Women with Osteoporosis - One-Year Results from the Dosing Intravenous Administration Study	Delmas, Pierre D	2006	Arthritis and Rheumatism	213	13.31 (42)
43	Elimination and Biochemical Responses to Intravenous Alendronate in Post-Menopausal Osteoporosis	Khan, Sohail A	1997	Journal of Bone and Mineral Research	194	7.76 (72)
44	Continuing Bisphosphonate Treatment for Osteoporosis - for Whom and for How Long?	Black, Dennis M	2012	New England Journal of Medicine	193	19.3 (26)
45	Fracture Risk and Zoledronic Acid Therapy in Men with Osteoporosis	Boonen, Steven	2012	New England Journal of Medicine	182	18.2 (29)
46	Risedronate Rapidly Reduces the Risk for Nonvertebral Fractures in Women with Post-Menopausal Osteoporosis	Harrington, JT	2004	Calcified Tissue International	180	10 (55)
47	Effect of Three Years of Oral Alendronate Treatment in Post-Menopausal Women with Osteoporosis	Tucci, Joseph R	1996	American Journal of Medicine	178	6.85 (77)
48	Effects of Teriparatide, Alendronate, or Both in Women with Postmenopausal Osteoporosis	Finkelstein, Joel S	2010	Journal of Clinical Endocrinology and Metabolism	177	14.75 (40)
49	Effects of Teriparatide and Alendronate on Vertebral Strength as Assessed by Finite Element Modeling of QCT Scans in Women with Osteoporosis	Keaveny, Tony M	2007	Journal of Bone and Mineral Research	174	11.6 (49)
50	A Systematic Review and Economic Evaluation of Alendronate, Etidronate, Risedronate, Raloxifene and Teriparatide for the Prevention and Treatment of Post-Menopausal Osteoporosis	Stevenson, M	2005	Health Technology Assessment	174	10.24 (53)
51	Dose-Response Relationships for Alendronate Treatment in Osteoporotic Elderly Women	Bone, Henry G	1997	Journal of Clinical Endocrinology and Metabolism	173	6.92 (76)
52	Additive Effects of Raloxifene and Alendronate on Bone Density and Biochemical Markers of Bone Remodeling in Post-Menopausal Women with Osteoporosis	Johnell, O	2002	Journal of Clinical Endocrinology and Metabolism	169	8.45 (65)
53	Effects of Long-Term Risedronate on Bone Quality and Bone Turnover in Women with Post-Menopausal Osteoporosis	Eriksen, EF	2002	Bone	165	8.25 (67)
54	Three Monthly Intravenous Injections of Ibandronate in the Treatment of Post-Menopausal Osteoporosis	Thiebaud, D	1997	American Journal of Medicine	164	6.56 (79)
55	Effects of Teriparatide and Risedronate on New Fractures in Post-Menopausal Women with Severe Osteoporosis (VERO): A Multicentre, Double-Blind, Double-Dummy, Randomized Controlled Trial	Kendler, David L	2018	Lancet	163	40.75 (10)
56	Benefits and Risks of Bisphosphonate Therapy for Osteoporosis	Khosla, Sundeep	2012	Journal of Clinical Endocrinology and Metabolism	162	16.2 (36)
57	Oral Alendronate Induces Progressive Increases in Bone Mass of the Spine, Hip, and Total Body over 3 Years in Post-Menopausal Women with Osteoporosis	Devogelaer, J. P	1996	Bone	161	6.19 (82)
58	Clinical Review: Bisphosphonate Use in Childhood Osteoporosis	Bachrach, Laura K	2009	Journal of Clinical Endocrinology and Metabolism	159	12.23 (69)
59	Two-Year Results of Once-Weekly Administration of Alendronate 70 mg for the Treatment of Post-Menopausal Osteoporosis	Greenspan, SL	2002	Journal of Bone and Mineral Research	159	7.95 (47)
60	Bisphosphonate-Related Osteonecrosis of the Jaw: Position Paper from the Allied Task Force Committee Of Japanese Society for Bone and Mineral Research, Japan Osteoporosis Society, Japanese Society of Periodontology, Japanese Society for Oral and Maxillofacial Radiology, and Japanese Society of Oral and Maxillofacial Surgeons	Yoneda, Toshiyuki	2010	Journal of Bone and Mineral Metabolism	157	13.08 (70)
61	Significant Differential Effects of Alendronate, Estrogen, or Combination Therapy on the Rate of Bone Loss after Discontinuation of Treatment of Post-Menopausal Osteoporosis - A Randomized, Double-Blind, Placebo-Controlled Trial	Greenspan, SL	2002	Annals of Internal Medicine	157	7.85 (43)
62	Alendronate or Alfacalcidol in Glucocorticoid-Induced Osteoporosis	De nijs, Ron N. J	2006	New England Journal of Medicine	151	9.44 (59)
63	Poor Bisphosphonate Adherence for Treatment of Osteoporosis Increases Fracture Risk: Systematic Review and Meta-Analysis	Imaz, I	2010	Osteoporosis International	150	12.5 (45)
64	Osteonecrosis of the Jaw and Bisphosphonate Treatment for Osteoporosis	Rizzoli, Rene	2008	Bone	149	10.64 (52)
65	Comparison of Teriparatide and Bisphosphonate Treatment to Reduce Pedicle Screw Loosening after Lumbar Spinal Fusion Surgery in Postmenopausal Women with Osteoporosis from a Bone Quality Perspective	Ohtori, Seiji	2013	Spine	148	16.44 (35)
66	Patient Preference for Once-Monthly Ibandronate versus Once-Weekly Alendronate in a Randomized, Open-Label, Crossover Trial: The boniva Alendronate Trial in Osteoporosis (BALTO)	Emkey, R	2005	Current Medical Research and Opinion	147	8.65 (64)
67	Cyclical Etidronate Reverses Bone Loss of the Spine and Proximal Femur in Patients with Established Corticosteroid-Induced Osteoporosis	Struys, Ard	1995	American Journal of Medicine	143	5.3 (88)
68	Addition of Alendronate to Ongoing Hormone Replacement Therapy in the Treatment of Osteoporosis: A Randomized, Controlled Clinical Trial	Lindsay, R	1999	Journal of Clinical Endocrinology and Metabolism	141	6.13 (83)
69	Maintained Improvement in Calcium Balance and Bone Mineral Content in Patients with Osteoporosis Treated with the Bisphosphonate APD.	Valkema, R	1989	Bone and Mineral	141	4.27 (99)
70	Risedronate Preserves Bone Architecture in Post-Menopausal Women with Osteoporosis as Measured by Three-Dimensional Microcomputed Tomography	Borah, B	2004	Bone	139	7.72 (73)
71	Efficacy and Safety of Alendronate for the Treatment of Osteoporosis in Diffuse Connective Tissue Diseases in Children - A Prospective Multicenter Study	Bianchi, ML	2000	Arthritis and Rheumatism	139	6.32 (81)
72	Changes in Bone Histomorphometry after Long-Term Treatment with Intermittent, Cyclic Etidronate for Post-Menopausal Osteoporosis.	Storm, T	1993	Journal of Bone and Mineral Research	139	4.79 (92)
73	Effect of Monitoring Bone Turnover Markers on Persistence with Risedronate Treatment of Post-Menopausal Osteoporosis	Delmas, Pierre D	2007	Journal of Clinical Endocrinology and Metabolism	137	9.13 (63)
74	Biochemical Markers Can Predict the Response in Bone Mass During Alendronate Treatment in Early Post-Menopausal Women	Ravn, P	1999	Bone	137	5.96 (86)
75	Early Responsiveness of Women with Osteoporosis to Teriparatide after Therapy with Alendronate or Risedronate	Miller, Paul D	2008	Journal of Clinical Endocrinology and Metabolism	136	9.71 (57)
76	Insufficiently Dosed Intravenous Ibandronate Injections are Associated with Suboptimal Antifracture Efficacy in Post-Menopausal Osteoporosis	Recker, R	2004	Bone	134	7.44 (74)
77	The Effect on Bone Mass and Bone Markers of Different Doses of Ibandronate: A New Bisphosphonate for Prevention and Treatment of Post-Menopausal Osteoporosis: A 1-Year, Randomized, Double-Blind, Placebo-Controlled Dose-Finding Study	Ravn, P	1996	Bone	130	5 (90)
78	The Effect of 6 versus 9 Years of Zoledronic Acid Treatment in Osteoporosis: A Randomized Second Extension to the HORIZON-Pivotal Fracture Trial (PFT)	Black, Dennis M	2015	Journal of Bone and Mineral Research	126	18 (30)
79	Effects of Oral Alendronate in Elderly Patients with Osteoporosis and Mild Primary Hyperparathyroidism	Rossini, M	2001	Journal of Bone and Mineral Research	125	5.95 (87)
80	Continuous Therapy with Pamidronate, a Potent Bisphosphonate, in Post-Menopausal Osteoporosis	Reid, IR	1994	The Journal of Clinical Endocrinology and Metabolism	125	4.46 (97)
81	A Four-Year Randomized Controlled Trial of Hormone Replacement and Bisphosphonate, Alone or in Combination, in Women with post-Menopausal Osteoporosis	Wimalawansa, Sunil J	1998	American Journal of Medicine	123	5.13 (89)
82	Effects of Oral Alendronate and Intranasal Salmon Calcitonin on Bone Mass and Biochemical Markers of Bone Turnover in Post-Menopausal Women with Osteoporosis	Adami, S	1995	Bone	123	4.56 (96)
83	Once-Weekly Risedronate in Men with Osteoporosis: Results of a 2-Year, Placebo-Controlled, Double-Blind, Multicenter Study	Boonen, Steven	2009	Journal of Bone and Mineral Research	120	9.23 (61)
84	Romosozumab (Sclerostin Monoclonal Antibody) versus Teriparatide in Post-Menopausal Women with Osteoporosis Transitioning from Oral Bisphosphonate Therapy: A Randomized, Open-Label, Phase 3 Trial	Langdahl, Bente L	2017	Lancet	118	23.6 (23)
85	Efficacy and Tolerability of Intravenous Ibandronate Injections in Post-Menopausal Osteoporosis: 2-year Results from the DIVA Study	Eisman, John A	2008	Journal of Rheumatology	117	8.36 (66)
86	Ibandronate in Osteoporosis: Preclinical Data and Rationale for Intermittent Dosing	Bauss, F	2004	Osteoporosis International	116	6.44 (80)
87	Intermittent Intravenous Ibandronate Injections Reduce Vertebral Fracture Risk in Corticosteroid-Induced Osteoporosis: Results from a Long-Term Comparative Study	Ringe, JD	2003	Osteoporosis International	116	6.11 (84)
88	Efficacy of Risedronate in Men with Primary and Secondary Osteoporosis: Results of a 1-Year Study	Ringe, JD	2006	Rheumatology International	115	7.19 (75)
89	Efficacy and Safety of a Once-Yearly i.v. Infusion of Zoledronic Acid 5 mg versus a Once-Weekly 70-mg Oral Alendronate in the Treatment of Male Osteoporosis: A Randomized, Multicenter, Double-Blind, Active-Controlled Study	Orwoll, Eric S	2010	Journal of Bone and Mineral Research	114	9.5 (58)
90	Clinic Visits and Hospital Admissions for Care of Acid-Related Upper Gastrointestinal Disorders in Women Using Alendronate for Osteoporosis.	Ettinger, B	1998	The American Journal of Managed Care	112	4.67 (95)
91	A Double-Masked Multicenter Comparative Study between Alendronate and Alfacalcidol in Japanese Patients with Osteoporosis	Shiraki, M	1999	Osteoporosis International	110	4.78 (93)
92	Incidence of Osteonecrosis of the Jaw in Women with Post-Menopausal Osteoporosis in the Health Outcomes and Reduced Incidence with Zoledronic Acid Once Yearly Pivotal Fracture Trial	Grbic, John T	2008	Journal of the American Dental Association	109	7.79 (71)
93	Comparison of Weekly Treatment of Post-Menopausal Osteoporosis with Alendronate versus Risedronate over Two Years	Bonnick, Sydney	2006	Journal of Clinical Endocrinology and Metabolism	107	6.69 (78)
94	Cyclical Etidronate in the treatment of Post-Menopausal Osteoporosis: Efficacy and Safety after Seven Years of Treatment	Miller, Paul D	1997	American Journal of Medicine	107	4.28 (98)
95	Denosumab or Zoledronic Acid in Postmenopausal Women with Osteoporosis Previously Treated with Oral Bisphosphonates	Miller, P. D	2016	Journal of Clinical Endocrinology and Metabolism	106	17.67 (68)
96	Evidence-Based Guidelines for the Use of Biochemical Markers of Bone Turnover in the Selection and Monitoring of Bisphosphonate Treatment in Osteoporosis: a Consensus Document of the Belgian Bone Club	Bergmann, P	2009	International Journal of Clinical Practice	106	8.15 (31)
97	Efficacy of Pamidronate for Osteoporosis in Patients with Cystic Fibrosis following Lung Transplantation	Aris, RM	2000	American Journal of Respiratory and Critical Care Medicine	105	4.77 (94)
98	Primary Prevention of Glucocorticoid-Induced Osteoporosis with Intravenous Pamidronate and Calcium: A Prospective Controlled 1-Year Study Comparing a Single Infusion, an Infusion Given Once Every 3 Months, and Calcium Alone	Boutsen, Y	2001	Journal of Bone and Mineral Research	104	4.95 (91)
99	Etidronate Disodium in Post-Menopausal Osteoporosis	Heaney, R P	1976	Clinical Pharmacology and Therapeutics	104	2.26 (100)
100	Alendronate, Vitamin D, and Calcium for the Treatment of Osteopenia/Osteoporosis Associated with HIV Infection	Mondy, K	2005	JAIDS (Journal of Acquired Immune Deficiency Syndromes)	102	6 (85)

**Table 2:** Journal with more than two of the 100 most-cited publications on bisphosphates for osteoporosis.

Journal	Article	Total citation	Mean citation	Impact factor
New England Journal of Medicine	16	10188	727.7	91.24
Journal of Bone and Mineral Research	16	3805	253.7	6.74
Journal of Clinical Endocrinology and Metabolism	14	2565	213.75	5.953
Bone	8	724	142.3	4.392
American Journal of Medicine	6	989	164.8	4.962
Osteoporosis International	5	1671	334.2	4.502
Arthritis and Rheumatism	4	866	216.5	10.991
Calcified Tissue International	3	658	219.3	4.331
Lancet	3	593	197.7	79.323
Annals of Internal Medicine	2	378	189	25.392
Current Medical Research and Opinion	2	468	234	2.58
Endocrine Reviews	2	549	274.5	19.871

**Table 3:** Authors with more than 5 articles in the 100 most-cited articles on bisphosphates for osteoporosis.

Author	No. of articles	First author	Last author	No. of total citations
Miller, PD	15	4	2	5831
Delmas, PD	13	2	4	5282
Reginster, JY	11	2	1	3040
Adami, S	9	1	1	2524
Boonen, S	9	2	2	3685
Felsenberg, D	8	0	0	2898
Greenspan, SL	8	2	0	2577
Black, DM	8	7	0	4793
Recker, RR	7	1	1	2151
Saag, KG	7	4	0	2683
Devogelaer, JP	7	1	0	2367
Cummings, SR	7	0	3	3602
Christiansen, C	6	0	2	1749
Eastell, R	6	0	3	3925
Eriksen, EF	6	1	1	4325
Lakatos, P	6	0	0	2856
Bone, HG	6	2	0	2071
Roux, C	6	0	0	2401
Bolognese, MA	5	0	0	878
Cosman, F	5	1	0	2688

**Table 4:** Type of study, patient, drugs, and complication of the 100 most-cited articles on bisphosphates for osteoporosis.

Article type	Number of articles
Original study	89
Clinic	87
Intervention trial	2
Systematic review/meta-analysis	5
Review	5
Editorial material	1
Patient	
Postmenopausal women	60
Glucocorticoid-induced patient	11
Men	6
Childhood	2
Bisphosphates drugs	
Alendronate	42
Risedronate	18
Ibandronate	11
Zoledronic	9
Etidronate	9
Complication	
Osteonecrosis of the jaw	3
Upper gastrointestinal disorders	1

## Data Availability

All data generated or analyzed during this study are included in this published article.

## Abbreviations

WOS: Web of Science
BMD: Bone mineral density
DXA: Dual x-ray absorptiometry.
CPYI: Citations per year index.
